# Optimization of steady‐state free precession MRI for lung ventilation imaging with ^19^F C_3_F_8_ at 1.5T and 3T

**DOI:** 10.1002/mrm.27479

**Published:** 2018-11-02

**Authors:** Adam Maunder, Madhwesha Rao, Fraser Robb, Jim M. Wild

**Affiliations:** ^1^ POLARIS, Unit of Academic Radiology, Department of IICD University of Sheffield Sheffield United Kingdom; ^2^ GE Healthcare Aurora Ohio; ^3^ Insigneo Institute for In silico medicine Sheffield United Kingdom

**Keywords:** fluorine‐19, Lungs, MRI, steady state free precession, ventilation

## Abstract

**Purpose:**

To optimize ^19^F imaging pulse sequences for perfluoropropane (C_3_F_8_) gas human lung ventilation MRI considering intrinsic in vivo relaxation parameters at both 1.5T and 3T.

**Methods:**

Optimization of the imaging parameters for both 3D spoiled gradient (SPGR) and steady‐state free precession (SSFP) ^19^F imaging sequences with inhaled 79% C_3_F_8%_ and 21% oxygen was performed. Phantom measurements were used to validate simulations of SNR. In vivo parameter mapping and sequence optimization and comparison was performed by imaging the lungs of a healthy adult volunteer. T_1_ and T_2_^*^ mapping was performed in vivo to optimize sequence parameters for in vivo lung MRI. The performance of SSFP and SPGR was then evaluated in vivo at 1.5T and 3T.

**Results:**

The in vivo T_2_^*^ of C_3_F_8_ was shown to be dependent upon lung inflation level (2.04 ms ± 36% for residual volume and 3.14 ms ± 28% for total lung capacity measured at 3T), with lower T_2_^*^ observed near the susceptibility interfaces of the diaphragm and around pulmonary blood vessels. Simulation and phantom measurements indicate that a factor of ~2‐3 higher SNR can be achieved with SSFP when compared with optimized SPGR. In vivo lung imaging showed a 1.7 factor of improvement in SNR achieved at 1.5T, while the theoretical improvement at 3T was not attained due to experimental SAR constraints, shorter in vivo T_1_, and B_0_ inhomogeneity.

**Conclusion:**

SSFP imaging provides increased SNR in lung ventilation imaging of C_3_F_8_ demonstrated at 1.5T with optimized SSFP similar to the SNR that can be obtained at 3T with optimized SPGR.

## 1 INTRODUCTION

MRI of lung ventilation with inhaled inert hyperpolarized (HP) gases has a proven sensitivity for the assessment of lung ventilation changes in obstructive airways disease.^1^ MRI with fluorinated gases (e.g., SF_6_, C_2_F_6_, and C_3_F_8_) shows promise as a complementary or alternative method for lung ventilation imaging, but in contrast to HP gas MRI, does not require additional polarization equipment.^2^ Additionally, fluorinated gases may be mixed with oxygen (O_2_) and continuously breathed, possibly allowing simpler investigation of dynamic lung physiology, such as the measurement of fractional ventilation by multi‐breath washout^3^, ^4^, ^5^ without the complication of gas depolarization observed with HP gas. Efforts to improve the quality of fluorinated gas ventilation MRI has been ongoing.^1^, ^2^, ^6^, ^7^ However, obtaining high‐resolution ventilation images with fluorinated gases at thermal equilibrium is challenging because of the low spin density, short T_2_^*^ and constrained imaging time.^7^

Past strategies of fluorinated gas MRI have focused on the use of short *TE* spoiled gradient (SPGR) sequences with *TR* relatively close to *T_1_*. This is due to two constraints: first, the relatively short T_2_^*^ of fluorinated gases in the lungs and, second, the specific absorption ratio (SAR) considerations at 3T (the most common field strength used for imaging of fluorinated gases to date)*_._* For SPGR imaging with the repetition time *TR«T_1_*, and where the acquisition time ($T_{\mathrm{aq}}$) is approximately that of T_2_^*^,^8^, ^9^ the SNR per‐unit‐time is nearly constant with *TR* due to the competing factors of averaging, $T_{\mathrm{aq}}$ and longitudinal recovery.^9^, ^10^ For example, *TR* values of 20 ms^11^, ^12^ for SF_6_ with *T_1_* < 2 ms,^13^ and 20 ms^14^ or 13 ms^12^ for C_3_F_8_ with *T_1_* ∼12.4 ms^14^ However, if *TR* is of the same order as *T_1_*, the optimization of single‐echo SPGR sequences generally requires minimizing *TR* so that the rate of longitudinal recovery for each *TR* is maximized.^10^

More recently, studies of C_3_F_8_ imaging have been performed at 1.5T using a 16‐element receive array.^15^, ^16^ However, imaging was still performed with *T_1_* ∼ *TR* *=* 12 ms, and a $T_{\mathrm{aq}}$ of 7.1 ms, which is significantly longer than the T_2_^*^. Therefore, future fluorinated gas imaging can clearly benefit from imaging parameter optimization as presented here.

In free‐gas phantoms, C_3_F_8_ gas has a longer *T_2_* (~17 ms 17) when compared with other fluorinated gases (∼4.2 for SF_6_ 18 and ∼5.9 for C_2_F_6_ 2), so improved signal to noise may potentially be achieved with the use of SSFP. The optimization of imaging parameters for SPGR^8^ and SSFP^19^ ^1^H MRI has been detailed previously. Also, the optimization of SSFP imaging parameters^20^ has been investigated for the imaging constraints of HP gas ventilation MRI with both ^3^He and ^129^Xe.^21^ Sequence optimization for perfluorocarbon emulsions has also been performed previously,^22^, ^23^ but in this instance the *T_2_* and *T_1_* relaxation parameters are significantly longer than for gas phase perfluorocarbons.

In this work, we demonstrate the application of SSFP sequences for ^19^F lung ventilation imaging using C_3_F_8_/O_2_ gas at 1.5T and 3T. Optimization of SSFP and SPGR imaging parameters was carried out by simulation with the specific relaxation parameters of C_3_F_8_/O_2_ gas as found in phantoms. The additional consideration of k‐space filtering^24^ from T_2_^*^ decay was explored by simulation of the 1D point spread function (PSF).^25^ Simulations of the SSFP signal were performed and compared experimentally with those achievable with a SPGR sequence. Constraints posed by SAR for in vivo applications are highlighted and the relaxation parameters *T_1_* and T_2_^*^ were mapped in vivo to verify the parameters used in simulation. Finally, in vivo lung imaging was performed with both sequences at 1.5T and 3T to test the theoretical/experimental predictions of SNR improvement. This study is to benchmark optimal imaging parameters.

## 2 THEORY

### 2.1 Simulations of SPGR and SSFP signal for C_3_F_8_

The two sequences considered here for 3D lung ventilation imaging with ^19^F perfluoropropane were SPGR and SSFP. For the SPGR sequence, transverse magnetization is de‐phased after each RF pulse by application of spoiling gradients.^26^ Conversely, in SSFP the phase of the excitation pulse phase alternates by ± π each *TR*, resulting in recycling of the transverse magnetization, while gradients refocus spins after acquisition for balanced SSFP (bSSFP).^27^

In the simulations presented, transverse magnetization ($M_{\mathrm{xy}})$ was evaluated at TE, which correlates with the center of k‐space and thus determines image signal intensity. Simulations of $M_{\mathrm{xy}}$ with SSFP were performed according to Hargreaves et al,^20^ with an effective transverse decay rate term of $T_{2}$.^28^ The steady state $M_{\mathrm{xy}}$ with SPGR, using the Ernst angle for maximum signal,^29^ was calculated as^8^, ^30^, ^31^,

(1) $$M_{\mathrm{xy}}=M_{0}\frac{e^{-TE/T_{2}^{*}}\left(1-e^{-TR/T_{1}}\right)\text{sin}\left(\alpha \right)}{1-e^{-TR/T_{1}}\text{cos}\alpha }=M_{0}\frac{e^{-TE/T_{2}^{*}}\left(1-e^{-TR/T_{1}}\right)}{\sqrt{1-e^{-2TR/T_{1}}}}$$

where α is the flip angle (FA).

For both SSFP and SPGR, the resulting image SNR is related to the transverse magnetization by^8^,

(2) $$SNR\alpha \frac{M_{\mathrm{xy}}}{M_{0}}\Delta V\sqrt{\frac{N_{\mathrm{avg}}}{\mathrm{BW}}}=\frac{M_{\mathrm{xy}}}{M_{0}}\Delta V\sqrt{\frac{T_{s}}{N_{p}}}\sqrt{\frac{T_{\mathrm{aq}}}{\mathrm{TR}}}$$

where BW is the bandwidth per‐pixel, $N_{\mathrm{avg}}$ is the number of averages, ΔV is the voxel size, $N_{p}$ is the number of phase‐encode steps, $T_{\mathrm{aq}}$ is the readout gradient acquisition time and $T_{s}$ is the total imaging time. The factor $T_{\mathrm{aq}}/TR$ represents the efficiency of the sequence in terms of maximizing the fraction of the *TR* devoted to sampling the signal. The expected optimal $T_{\mathrm{aq}}$ with SPGR is close to $T_{\mathrm{aq}}\approx T_{2}^{*}$.^9^ For the sake of a fair SSFP and SPGR comparison, the spatial resolution, imaging time, and *y* and *z* phase encoding steps remained the same. The effects of SSFP signal transient behavior on the final SNR were ignored, which was justified by the relatively short *T_1_* and *T_2_* when compared with *TR*, resulting in a steady state being reached rapidly.

With HP ^3^He gas it has been demonstrated that dephasing from the imaging gradients has a significant effect on the effective transverse relaxation rate,^24^ while the effect is less significant when imaging with ^129^Xe because of the much lower diffusion coefficient.^32^ Calculations with the even lower diffusion coefficient of C_3_F_8_,^17^ with its relatively low *T_2_*, indicate that this effect is small when compared with the uncertainty/variability in the *T_2_* and, therefore, the effect of diffusion dephasing due to the imaging gradients themselves was neglected here.

Imaging timing parameters that impact upon *TE* and $T_{\mathrm{aq}}$ include the following: the RF pulse width ($T_{\mathrm{pw}}$) and imaging gradient encoding/refocusing delays before ($T_{D1}$) and after ($T_{D2}$) frequency encoding. Therefore, *TE* = $\frac{T_{\mathrm{pw}}}{2}+T_{D1}+\frac{T_{\mathrm{aq}}}{2}$ and $T_{\mathrm{aq}}=TR-T_{\mathrm{pw}}-T_{D1}-T_{D2}$. To emulate practical imaging sequence timings, the simulated RF pulse widths were matched to the measurement values, while $T_{D1}$ and $T_{D2}$ were selected to be 0.6 ms throughout the comparison to closely match those used in measurement.

### 2.2 Quantification of *T* *_2_* ^*^ decay induced *k* *_x_* filtering

Insight into the reduction in image quality due to $T_{2}^{*}$ filtering during frequency encoding (*k_x_*) was attained by comparison of the 1D PSF of the different sequences. For SPGR the signal decays exponentially from the center of the RF excitation pulse with a time constant T_2_^*^. For SSFP the signal is modeled as decaying exponentially with time constant *T_2_*, as well as decaying symmetrically away from *TE* with the time constant T_2_^*^, similar to simulation/measurement performed in reference with a spin‐echo sequence,^33^ as the transverse magnetism ideally decays similarly in a bSSFP sequence.^28^

### 2.3 Relaxation parameters of C_3_F_8_/O_2_

For the phantom simulations presented here the *T_1_* and *T_2_* of C_3_F_8_ gas mixed with 21% O_2_ are assumed to be 17 ms^17^ Within the lung the *T_1_* of fluorinated gases is known to depend more upon regional differences in partial‐pressure^34^, ^35^ of O_2_. Consequently, the mean in vivo *T_1_* has been reported as 12.4 ms at 3T.^14^ Additionally, the intrinsic *T_2_* of C_3_F_8_ gas within the lungs has not been reported, but is expected to remain comparable to *T_1_*.^17^, ^34^ Additionally, the mean in vivo T_2_^*^ relaxation constant of C_3_F_8_ has been reported as ∼2.2 ms at 3T.^14^

## 3 METHODS

### 3.1 Simulation of steady‐state magnetization with SSFP

The relation between steady‐state magnetization, *FA*, and RF frequency offset from resonance were simulated with MATLAB considering the particular relaxation parameters of C_3_F_8_ for 3D imaging with a *TR* of 3.4 ms. Additionally, to assess whether transient oscillations in the magnetization during initial RF excitations are significant, the transverse magnetization for successive RF excitations was simulated for different values of *TR* Furthermore, to quantify the expected 1D PSF arising from transverse magnetization decay the PSF was simulated for varying *T_aq_*.

### 3.2 Validation of simulated magnetization with phantom SNR measurements

To compare the simulations of signal for C_3_F_8_ for SSFP versus SPGR, phantom experiments were carried out with a 2‐L glass cylinder (12 cm diameter, 20 cm length) filled with 79% C_3_F_8%_ and 21% O_2_ at 1.4 bar pressure. Rectangular (24 cm × 16 cm) transceive single loop coils were constructed from 11 mm width copper strip, tuned and matched at the 1.5T (GE Signa HDx) (60 MHz) and 3T (Philips Ingenia) (120 MHz) frequencies and centered with the cylinders during imaging. Before the phantom studies at 1.5T and 3T, *FA* maps were generated by varying the input power in SPGR imaging with *TR* 100 ms» *T_1_* and fitting the received signal according to Equation (1), as in Maunder et al.^36^ The prescribed *FA* recorded in Table 1 for the imaging performed with the glass phantoms was based on the fitted FA at the center of the phantom. Furthermore, to ensure that SNR and relaxation parameters were not inaccurately calculated due to B_1_ inhomogeneity, voxelwise parameter mapping was calculated using the voxelwise fitted FA map^37^ rather than a prescribed mean value.

**Table 1:** Imaging parameters for phantom and in vivo SSFP and SPGR performance verification with C_3_F_8_

Measurement	Sequence	TE (ms)	TR (ms)	BW (± kHz)	Matrix size (pixels^3^)	FOV (cm^3^)	Prescribed FA (°)	Avg.	T_pw_ (µs)
1.5 T									
Phantom study									
SNR vs. FA	3D SPGR	1.6	4.3	10	50x50x10	20x20x16	13−91	10	468
SNR vs. FA	3D SSFP	1.6	3.9	10	50x50x10	20x20x16	18−120	10	616
FA mapping	3D SPGR	6.8	100	2.0	52x52x12	24x24x12	9−103	1	1600
T_1_ mapping	3D SPGR	1.4	5	12.5	52x52x12	24x24x12	8.5−52	40	1600
T_2_^*^ mapping	2D SPGR	1.5−11	250	31.25	52x52x12	24x24x12	47	2	1600
SNR vs. TR	3D SPGR	6.8−0.7	15.4−3.2	2−62.5	52x52x12	24x24x12	66−34	5	1600
SNR vs. TR	3D SSFP	6.8−0.7	15.4−3.2	2−62.5	52x52x12	24x24x12	89.6	5	1600
In vivo comparison									
FA mapping	3D SPGR	2.2	35	3.97	32x26x10	40x32x30	27.5/55/82	1	832
T_1_ mapping	3D SPGR	2.2	5.7	3.97	32x26x10	40x32x30	27.5/55/82	10	856
Optimal SNR comparison	3D SSFP	1.7	4.0	5.21	32x27x18	40x32x36	72	4	616
Optimal SNR comparison	3D SPGR	1.7	4.0	5.21	32x27x18	40x32x36	45	4	468
Ventilation image	3D SSFP	1.72	4.0	6.76	40x34x32	40x32x32	72	8	616
3 T									
Phantom study									
FA mapping	3D SPGR	6.4	100	3.1	52x52x12	24x24x12	10.6−85	1	1600
T_1_ mapping	3D SPGR	2.1	5	22.6	52x52x12	24x24x12	22−82	10	1600
T_2_^*^ mapping	3D SPGR	1−30	80	45.1	52x52x12	24x24x12	42.5	5	1600
SNR vs. TR	3D SPGR	6.9−1.8	13−4.0	3.1−35.3	52x52x12	24x24x12	57.5−35	5	1600
SNR vs. TR	3D SSFP	7.2−2.1	13−4.0	3.1−32.2	52x52x12	24x24x12	85	5	1600
SNR vs. offset frequency	3D SSFP	2.1	4.6	12.2	50x50x5	20x20x10	22.5/75	10	1600
In vivo comparison									
FA mapping	3D SPGR	1.48*	50	4.3	28x27x12	40x40x24	30/90	2	1350
T_1_ mapping	3D SPGR	‐	6.5	4.3	28x27x12	40x40x24	25/37.5/50	5	1350
T_2_^* ^mapping	3D SPGR	1.0−6.0	7	46.3	32x29x14	40x35x29.3	26	12	1350
Optimal SNR comparison	3D SPGR	1.8	4	9.5	40x32x28	40x32x28	30	4	780
Optimal SNR comparison	3D SSFP	1.8	4	9.5	40x32x28	40x32x28	30	4	780
Ventilation image	3D SSFP	1.8	4	9.5	40x32x28	40x32x28	30	8	780

The assumed *T_1_* and *T_2_* relaxation parameters were verified by comparing the variation of image SNR and simulated steady‐state transverse magnetization with RF excitation frequency offset. The offset frequency was varied from −1/*TR* to 1/*TR* (*TR* = 4.6 ms) in steps of 30 Hz with two FAs (22.5° and 75°) and the SNR was evaluated within a central voxel of the glass cylinder phantom at 3T. Furthermore, the simulated transverse magnetization of SPGR and SSFP sequences were compared with measured image SNR with varying FA. The image SNR was averaged within a central 1.2 × 1.2 × 3 cm^3^ voxel with either 3D SSFP or SPGR imaging at 1.5T.

The restrictions on FA due to regulatory SAR contraints,^38^ when applying the same imaging sequence in vivo with a thoracic vest transceiver coil 36, 39 were also considered in the SPGR and SSFP SNR versus *FA* comparison. The vest transceiver coil is similar in geometry to the one used here and should have comparable SAR characteristics. For a 1 kW RMS input power, the maximum local 10 g averaged SAR was simulated within a realistic human body model (SIM4LIFE Zurich Med Tech, Duke model^40^) as 125 W/kg, with a 11.8 $\mu T/\sqrt{\mathrm{kW}}$ transmit efficiency at 60 MHz. The global SAR was calculated conservatively as the input power to the coil being completely deposited into a 70 kg patient. A constant 500 μs hard pulse width was assumed, while pulse amplitude was varied to match the *FA*. All simulated *FA*s were, therefore, achievable with the 4 kW peak power amplifier used in in vivo imaging at 1.5T. The specific imaging parameters for these and the in vivo imaging experiments detailed later are provided in Table 1.

### 3.3 Phantom relaxation parameters

The T_2_^*^ of C_3_F_8_ within the glass cylinder phantoms is not representative of in vivo values measured in the lung where tissue‐airspace field inhomogeneity plays a significant role. Therefore, a spatially varying T_2_^*^ inhomogeneity was introduced by placing a paramagnetic wire in close proximity to the glass canister. At 1.5T, T_2_^*^ maps were calculated by varying the *TE* in multiple image acquisitions, while fixing the *BW*, *FA*, and *TR*, then fitting according to Equation (1). The range of *TE* available at 1.5T was not high enough to accurately distinguish between T_2_^*^ values > 14 ms. However, at 3T, T_2_^*^ maps were fit from the signal decay during multi‐echo SPGR imaging (multiple echoes per *TR*) with *TE*s up to 30 ms. To determine that the paramagnetic inhomogeneity did not alter the *T_1_* relaxation parameter, and that the in‐phantom *T_1_* agreed with previous literature,^17^ *T_1_* was mapped throughout the cylinder by varying the *FA*, with a short *TR* (5 ms at 3T and 1.5T) and fitting pixel‐wise according to Equation (1).^41^

### 3.4 Simulated and measured optimization of SPGR and SSFP imaging parameters

To determine the optimal *TR* for 3D SPGR and SSFP imaging sequences, measurements were performed at 1.5T and 3T with varying *TR*. The same *FA* was used for SSFP imaging (approximately 90°), while the input RF power was varied with SPGR imaging to maintain the optimal Ernst *FA* at the center of the phantom. Three different regions of interest covering a range of T_2_^*^values were investigated.

The simulated steady‐state transverse magnetization was multiplied by the factor $\sqrt{\frac{T_{\mathrm{aq}}}{\mathrm{TR}}}$ to represent the SNR per‐unit‐time efficiency due to trade‐off between acquisition bandwidth and averaging. The simulated magnetization and measured SNR were plotted against *TR* The previously acquired FA maps were used to verify that within the representative voxels the difference in SNR due to potential mismatch in prescribed FA and optimal FA was less than 5%.

### 3.5 In vivo relaxation parameter mapping

In vivo lung ventilation imaging was performed in a healthy male adult volunteer (29 years old) following informed consent and adhering to protocols approved by UK National research ethics committee. An eight‐element in‐house constructed transceive array was used for 1.5T ^19^F and ^1^H in vivo imaging.^42^ An elliptical birdcage coil (Rapid Biomedical, Rimpar, Germany) was used for ^19^F and ^1^H imaging at 3T. The global FA was measured before imaging by performing whole‐lungs spectroscopy with a varying input power and long *TR* (268 ms at 1.5T and 200 ms at 3T) and then fitting the resulting signal according to Equation (1). Saturation of the lungs with the C_3_F_8_/O_2_ mixture was achieved by directing the volunteer to take three inhalations from a Douglas bag then perform a breath‐hold.

To compare the global and regional variation of *T_1_* in vivo at 1.5T and 3T with that obtained in phantoms at 3T, *T_1_* and FA parameter mapping was performed. In the same breath‐hold two 3D SPGR imaging sequences were performed with a long *TR* relative to *T_1_* (*TR* = 50 ms at 3T and *TR* = 35 ms at 1.5T) and prescribed mean *FA*s of ∼90° *FA* and ∼30° *FA* (for 1.5T an additional point of ~60° was included). The resulting pixel‐wise FA was calculated based on the signal intensity difference according to Equation (1).^43^ In a second breath‐hold, three 3D SPGR imaging sequences were performed with *TR* shorter than *T_1_* (*TR* = 6.5 ms at 3T and *TR* = 5.7 ms at 1.5T) and the resulting pixel intensity variation used to fit T_1_.^44^

In addition, at 3T, T_2_^*^ mapping was performed to corroborate the presumed values. A multi‐echo SPGR acquisition was made with *TE* in the range of 1‐6 ms in 1‐ms steps, and the resulting images were fit on a voxel by voxel basis. T_2_* mapping was performed at the two lung volumes of total lung capacity (TLC) and residual volume (RV).

### 3.6 In vivo comparison of SPGR and SSFP image SNR

At 1.5T, SNR comparisons were made between a SPGR sequence with an approximately optimal prescribed *FA* (∼45° with *TR* = 4 ms), and a 3D SSFP imaging sequence with ∼70° FA, which was limited due to SAR constraints. At 3T, SPGR and SSFP imaging were performed with nearly identical imaging parameters, because SAR constraints restricted the *FA* to 30° with a *TR* of 4 ms The direct comparison of SPGR and SSFP sequences at each field strength was carried out within the same breath‐hold to avoid inconsistencies in coregistration or possible differences in the PFP: air concentration ratio in the lungs that may arise between breath‐holds (20 s at 1.5T and 28 s at 3T plus inter‐scan delay of approximately 5 s). Images were obtained with fully optimized sequences using the same resolution at both 1.5T and 3T for final comparison. Finally, to accurately compare the imaging methods k‐space was filtered with an identical Hamming filter before FFT reconstruction.^11^ As a final comparison between the two field strengths, imaging was performed at 1.5T and 3T with the same resolution (10 × 10 × 10 mm^3^) *TR* (4 ms) and 8 averages. To reduce the breath‐hold time, four averages were obtained in two separate breath‐holds to total lung capacity (20 s at 1.5T and 14 s at 3T which included an elliptical shutter).

## 4 RESULTS

### 4.1 Simulations for Informing Experimental Optimization

**Figure 1:**
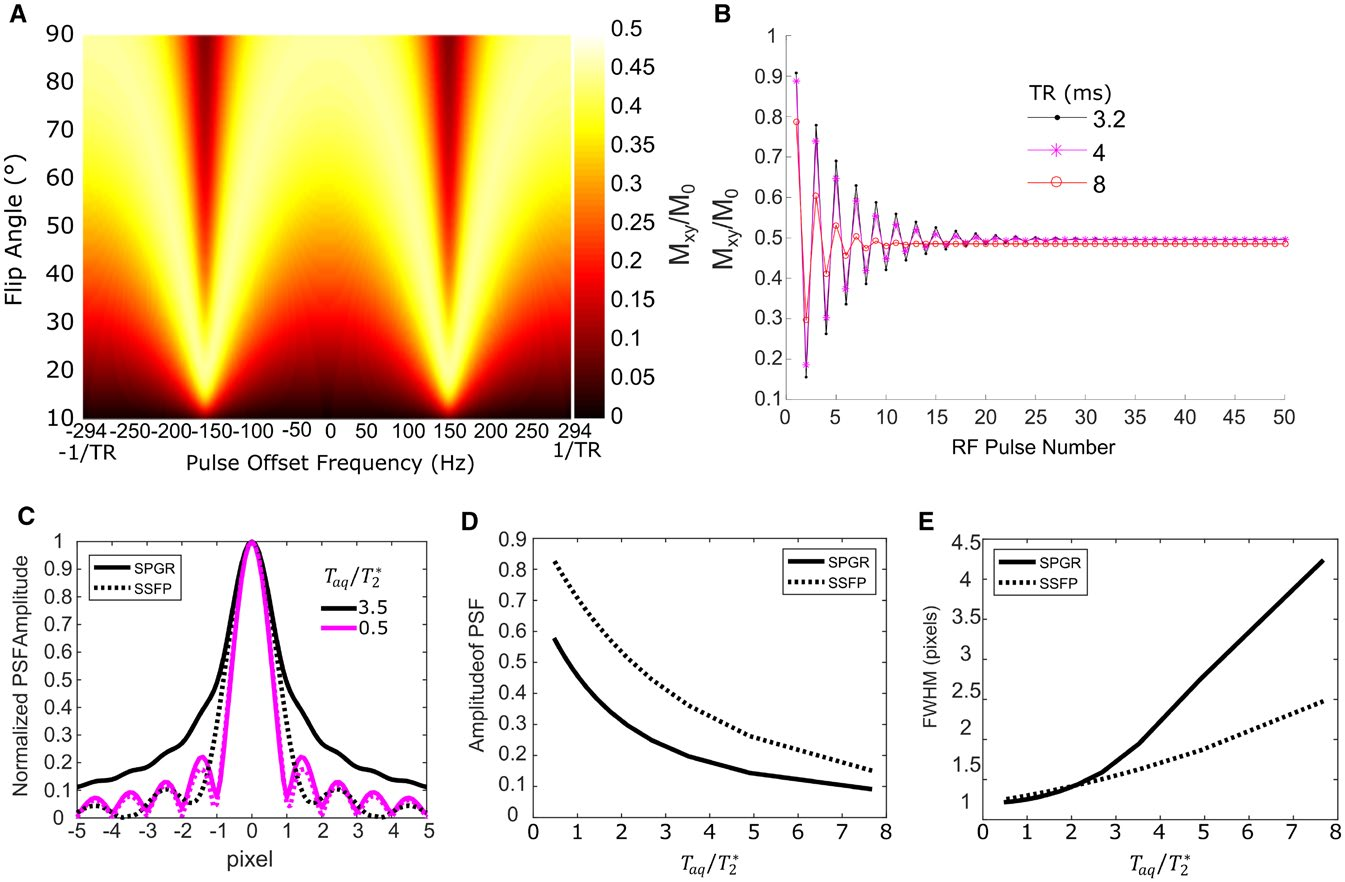
A, Simulated steady state magnetization as a function of FA and offset frequency for *TR* = 3.4 ms. B, Simulated transverse magnetization evolution for successive RF pulses (effective k_y_/k_z_ filter) for a bSSFP sequence with C_3_F_8_. C, The simulated normalized 1D PSF in the k_x_ direction from T_2_^*^ decay for both SSFP and SPGR sequences with relaxation parameters of C_3_F_8_. D, The corresponding simulated PSF amplitudes and E, FWHMs of PSFs with increasing T_aq_

Simulation of the C_3_F_8_ phantom steady state transverse magnetization with a *TR* = 3.4 ms is shown in Figure 1A, with varying FA and RF excitation offset frequency. Because *T_1_* is approximated as *T_1_* *=* *T_2_*, the transverse decay is equal to the longitudinal recovery rate and the optimal FA remains 90° for the central (0 Hz) offset frequency in all cases.^27^

The simulated oscillating transverse magnetization during the initial series of excitations is shown in Figure 1B for varying *TR* The rapid longitudinal recovery of C_3_F_8_ means that a steady‐state is reached within a short number of RF pulses for the *TR*s shown, reducing the amount of *k_y_* & *k_z_* filtering to a negligible level when SSFP imaging with C_3_F_8_.^24^ Therefore, the application of 10 stabilization excitations before imaging performed in this study reduced the variation in magnetization with subsequent RF pulse excitations to less than 10%, even for a relatively short *TR* of 3.2 ms.

The simulation of the 1D PSF during frequency encoding readout is shown in Figure 1C for both SPGR and SSFP. The resulting amplitudes of the PSFs for the different sequences is also shown in Figure 1D, and the FWHM of the PSF in Figure 1E. SPGR is deficient in terms of lower PSF amplitude and increased FWHM when compared with the SSFP PSF as the $T_{\mathrm{aq}}$ is increased. However, if $T_{\mathrm{aq}}$ is kept short relative to the T_2_^*^ the FWHM remains low and blurring is minimal. For SPGR and SSFP sequences with C_3_F_8_ if $T_{\mathrm{aq}}$ < *2* *T* *_2_* ^*^ the FWHM of the PSF remains comparable.

### 4.2 Simulation investigation and validation

**Figure 2:**
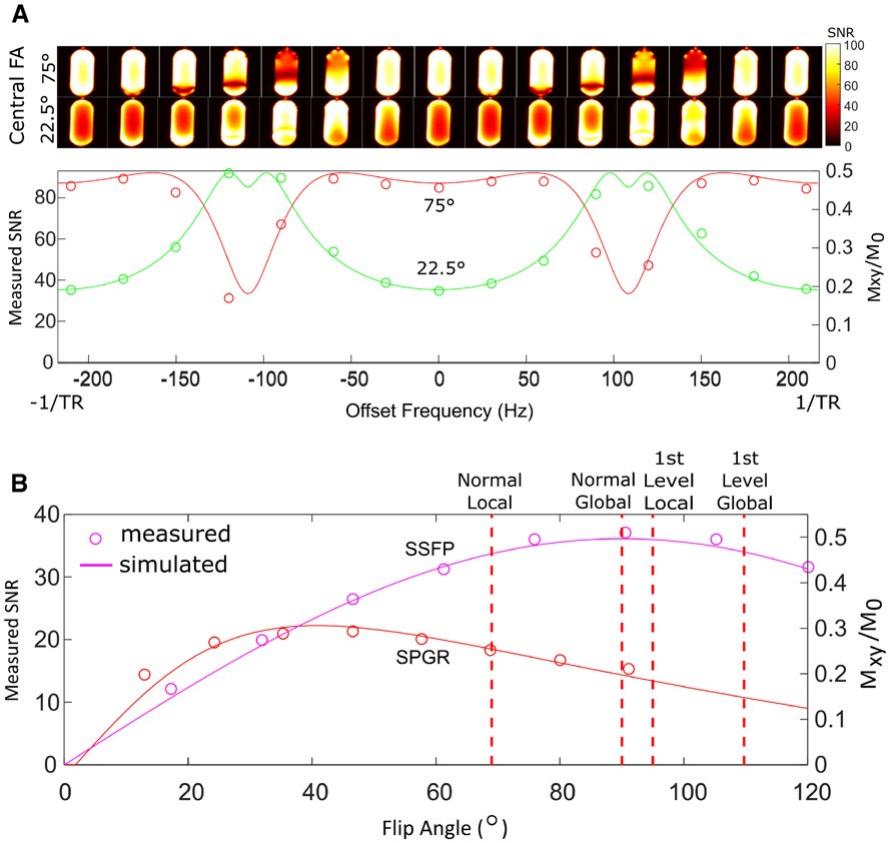
A, Simulated SSFP transverse magnetization and measured SNR versus offset frequency at 3T for a central ROI within the C_3_F_8_ gas phantom at 3T with *TR* of 4.3 ms SNR maps of a central slice are shown (above) as the offset frequency is varied for both 22.5º and 75º *FA*s. B, Simulated steady state magnetization and measured SNR at 1.5T in a central ROI of the PFP cylinder with 3D SPGR and SSFP sequences plotted as a function of varying *FA* demonstrating the close relation with simulation. Red dotted vertical lines indicate the calculated SAR limits based on *FA* if the same sequence were performed in vivo at 1.5T

Figure 2A shows the measured SNR of the SSFP signal at 3T with varying offset excitation frequency. As expected, the simulated magnetization displays a similar trend versus offset frequency when compared with measurement. Central slices are displayed for the varying offset frequency, demonstrating the introduction of banding artifacts arising from field inhomogeneity as the excitation frequency is offset from the center. In Figure 2B the relation between SNR and *FA* for both SPGR and SSFP sequences is demonstrated at 1.5T for a central region of interest (ROI) of the phantom. Here, a close relation between SNR and the simulated steady‐state magnetization is demonstrated, further validating the values of the relaxation parameters used in the simulations. The calculated SAR levels are displayed showing that a $90^{\circ }$ *FA* could be used within 1st level controlled SAR constraints. However, to maintain more conservative local SAR levels, FA < $70^{\circ }$ should be used for the specific *TR* and pulse width presented in this case.

### 4.3 C_3_F_8_ phantom SSFP versus SPGR SNR comparison

**Figure 3:**
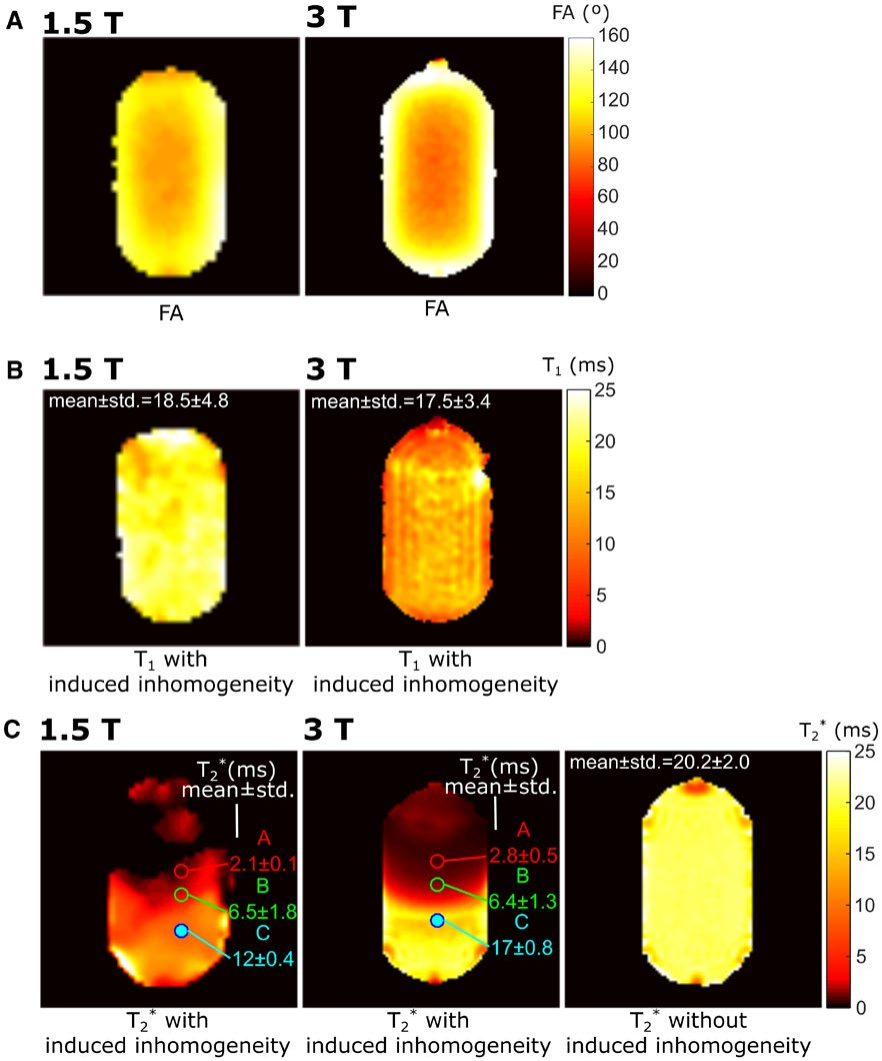
A, FA maps through a central slice at 1.5T and 3T. B, *T_1_* maps through a central slice are displayed with the placement of a paramagnetic wire at 1.5T and 3T. C, T_2_^*^ maps are also displayed for 1.5T and 3T originating from the placement of the paramagnetic wire. The T_2_^*^ map without the variation from the paramagnetic wire is shown for 3T as well. ROIs where SNR variation is evaluated as TR is varied and the corresponding T_2_^*^ for specific locations are displayed with the T_2_^*^ maps

Maps of the FA homogeneity that all subsequent phantom parameter mappings are based upon are displayed in Figure 3A. The *T_1_* map for the phantom at 1.5T and 3T is displayed in Figure 3B, and is in agreement with the range reported in Chang and Conradi^17^ at 60 MHz. The *T_1_* is expected to increase slightly with Larmor frequency (<1 ms larger at 176 MHz versus 60 MHz reported in Chang and Conradi^17^). Here, the standard deviation in the measurement was greater than the expected increase from 1.5T to 3T. The *T_1_* maps do not show any regional variation with proximity to the paramagnetic wire. In Figure 3C the T_2_^*^ maps for a central slice of the phantom with the paramagnetic wire added at both 1.5T and 3T. The T_2_^*^ map measured at 3T when the wire is excluded is also shown.

**Figure 4:**
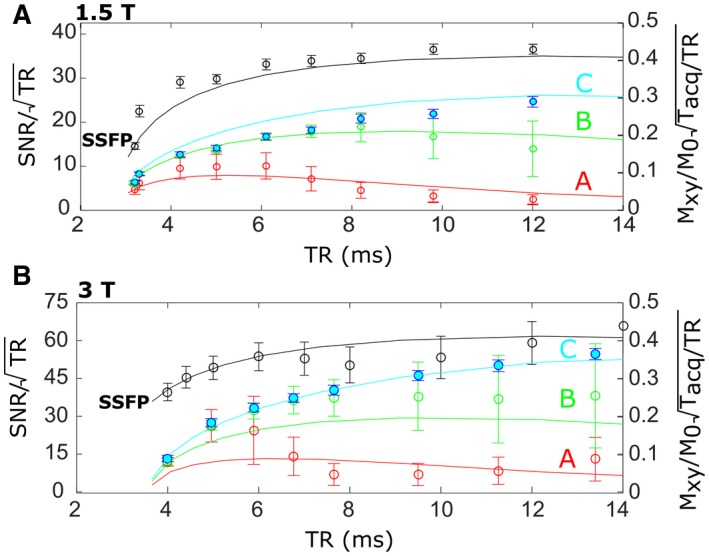
The measured variation (circular markers) of SNR with TR for SSFP (black) or SPGR (blue, green and red) sequences at A, 1.5T or B, 3T are displayed. For SPGR, labels of A, B, and C correspond to the ROIs in T_2_^*^ maps labelled similarly in Figure 3C. Image SNR is normalized by the time for averaging ($\sqrt{\mathrm{TR}}$), while the simulated transverse magnetization (solid lines) is normalized by the predicted *T_acq_* and *TR*

The main comparison of image SNR obtained with SPGR and SSFP sequences is displayed for varying *TR* in Figure 4A (at 1.5T) and Figure 4B (at 3T). The central ROIs were chosen to demonstrate the SNR variation with T_2_^*^ and are displayed on the T_2_^*^ maps in Figure 3C. As *TR* is varied the measured SNR remains significantly higher for SSFP when compared with SPGR. SPGR optimization is highly dependent on T_2_^*^, with maximal SNR occurring when the $T_{\mathrm{aq}}$ is slightly greater than T_2_^*^.

The simulated transverse magnetization (normalized for the time available time for acquisition and averaging) closely matches the measured ROI SNR. However, because the pixel ROIs include a range of T_2_^*^ the SNR behavior with TR does not match exactly. In simulation, the signal was assumed to correspond to the transverse magnetization amplitude at *k_x_* = 0 (center of the frequency encoding gradient), but in fact, is also dependent on the PSFs as presented in Figure 1D.

### 4.4 In vivo parameter mapping

**Figure 5:**
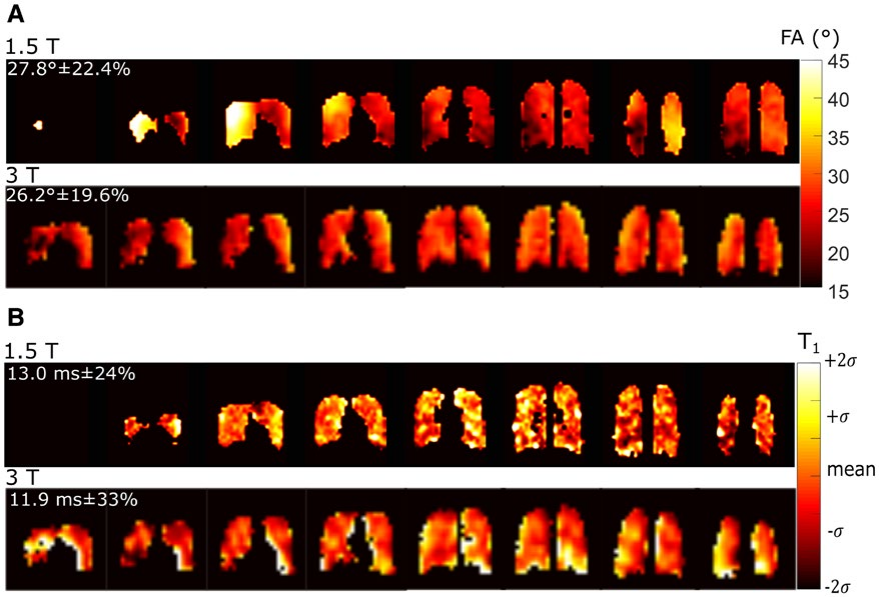
Maps of A, FA for a prescribed 30° and B, *T_1_* at 1.5T, for images acquired at a lung volume of TLC in a healthy volunteer. Parameter mapping results where the image SNR was < 20 were excluded from analysis. Maps include the mean and standard deviation of parameters throughout the lungs in the top left corner

Mapping of FA is displayed in Figure 5A, and the corresponding colocalized *T_1_* map in Figure 5B. The mean *T_1_* is lower than that found in the phantom (Figure 3), which is in agreement with previously reported in vivo *T_1_* from whole lungs (12.4 ms at 3T^14^). Regional variation is apparent, with the greatest variation observed at the lung‐tissue interfaces.

**Figure 6:**
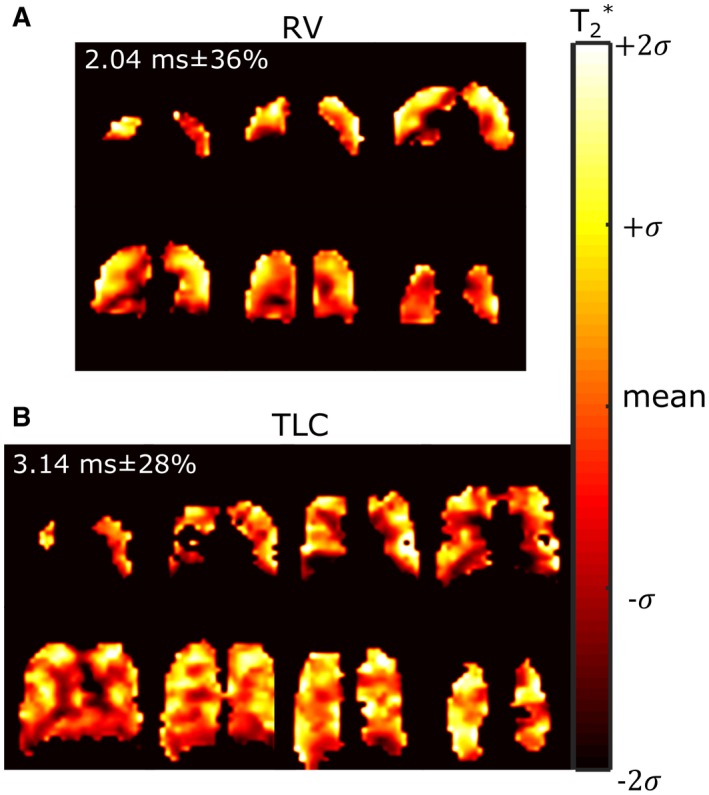
Maps of T_2_^*^ in vivo at 3T for lung volumes of A, RV and B, TLC are displayed with the mean and standard deviation of parameters throughout the lungs in the top left corner

T_2_^*^ maps are shown in Figure 6A and Figure 6B for lung inflation levels TLC and RV, respectively. The in vivo T_2_^*^ is systematically less than in the glass cylinder phantoms (average of 2.04 ms versus 20 ms in the phantom with undistorted field). The average T_2_^*^ at 3T is in agreement with previous global measurements for the PFP T_2_^*^ in the lungs (2.2 ms^14^), but the regional variation and dependence on inflation level is significant. There does not appear to be a visually observable correlation between regions of varying T_2_^*^ and *T_1_*, while T_2_^*^ seems to be lowest in regions near the susceptibility interfaces of the pulmonary blood vessels and at the inferior portion of lung where perfusion is highest.

### 4.5 In vivo SNR performance: SSFP versus SPGR

**Figure 7:**
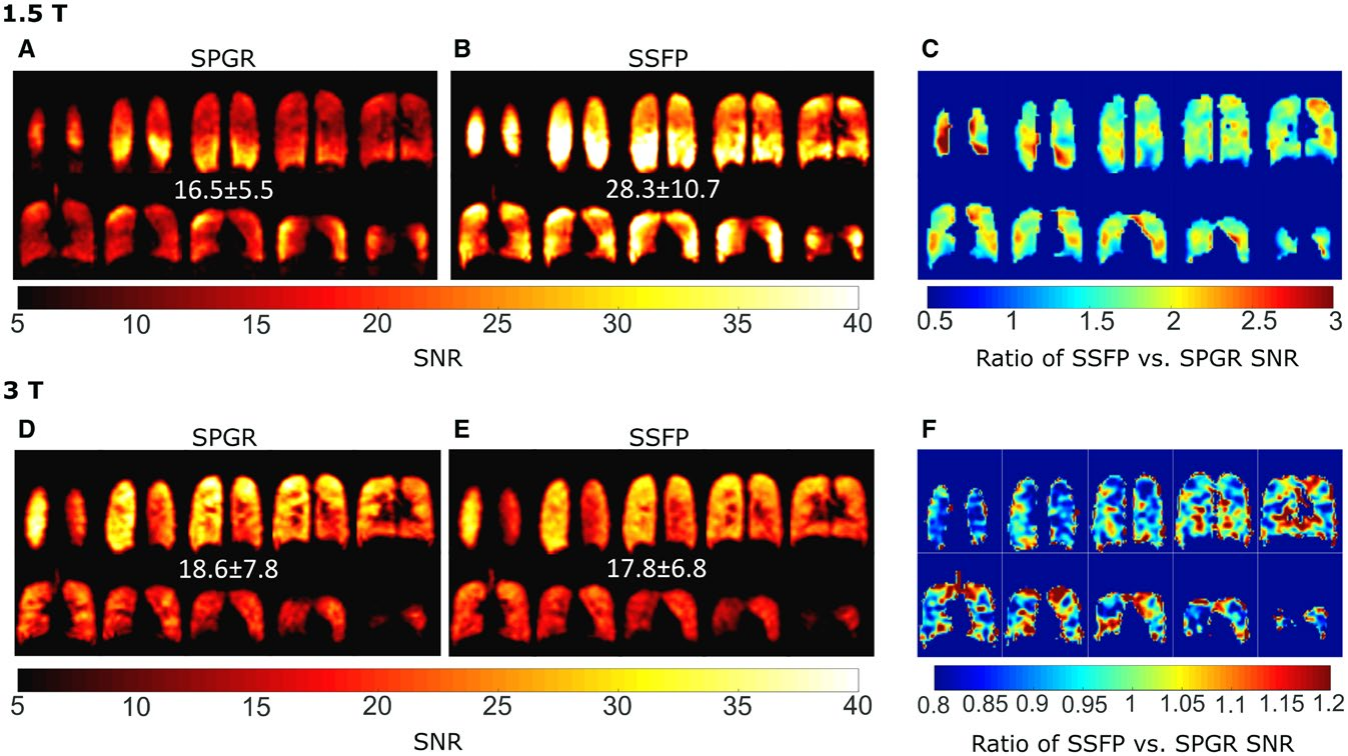
SNR maps of PFP in the lungs acquired at 1.5T using either A, SPGR or B, SSFP imaging and C, the relative improvement in SNR with SSFP imaging. Additionally, SNR maps acquired at 3T using D, SPGR or E, SSFP sequences with (F) maps of the relative ratio of SNR of SSFP versus SPGR imaging

An average increase in SNR by a factor of 1.7 was found at 1.5T (Figure 7A compared with Figure 7B). However, there are some bands of high versus low increases in SNR (Figure 7C) demonstrating the possible impact of field inhomogeneity. At 3T, no overall increase in SNR was observed with SSFP when performed under the SAR conservative settings (FA of $30^{\circ }$ and *TR* of 4 ms) when compared with SPGR imaging (Figure 7C compared with Figure 7D). The significant regional variation in the SNR increase throughout the lungs with SSFP versus SPGR is reflective of the local B_0_ inhomogeneity.

### 4.6 In vivo ventilation imaging: 1.5T and 3T comparison

**Figure 8:**
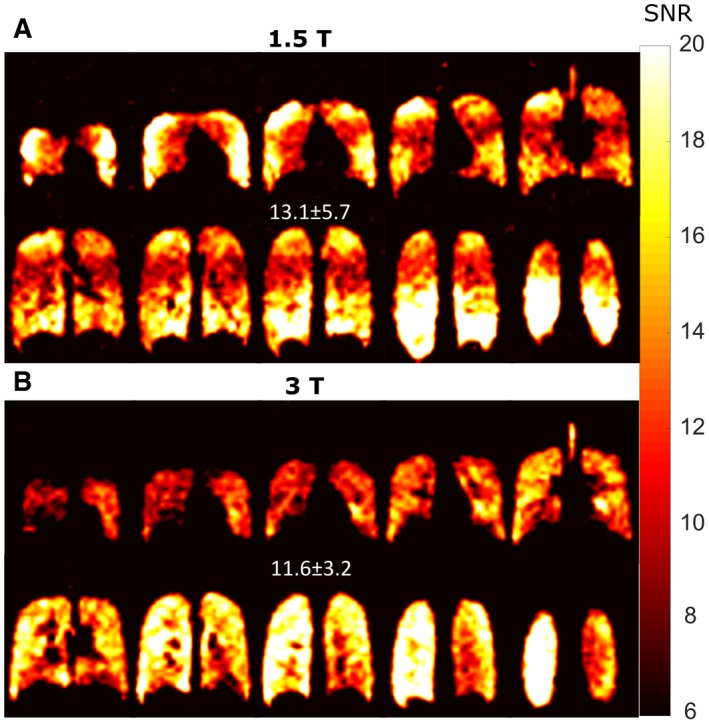
SNR maps for fully optimized imaging at equal resolution A, with SSFP imaging at 1.5T and an 8‐element array or B, at 3T with SPGR and a quadrature birdcage coil are shown for final 1.5T and 3T comparison

SNR maps of the in vivo ventilation images obtained at 1.5T and 3T are shown in Figure 8. Through the use of a transceive array and increased SNR with SSFP imaging the mean SNR at 1.5T is higher than that of 3T for the same resolution. The increase in SNR is dominated by the regions of increased coil sensitivity at the anterior and posterior regions of the lung and much of the periphery, so the variation is higher at 1.5T as well. Due to B_1_ inhomogeneity in the anterior of the lung at 3T, there is significant signal drop‐out.

## 5 DISCUSSION

The close agreement between the simulated and measured SSFP versus SPGR signals, with both varying FA and offset frequency, indicates that the expected parameters of *T_2_*, *T_1_*, and T_2_^*^ within the glass phantom are valid. Additionally, the direct measurement of *T_1_* and T_2_^*^ matched the expected in‐phantom values, with measurably smaller mean values of *T_1_* and T_2_^*^ measured in vivo. *T_2_* was indirectly validated by the close agreement between SSFP simulations and measurements because measuring *T_2_* with established spin echo sequences was constrained by the SAR limitations. For short sequence *TR*, variations in the simulated *T_2_* and *T_1_* for C_3_F_8_ have minimal influence on the simulated steady‐state magnetization, because they are expected to remain comparable.^17^ However, lower *T_1_* results in a predicted greater steady‐state magnetization with SPGR. This manifests as a reduction in the relative improvement of SSFP imaging of PFP in the lungs when compared with in a PFP gas phantom, which was observed at both 1.5T and 3T. It was also demonstrated that the improved SNR achieved using SSFP when compared with SPGR is strongly dependent upon the T_2_^*^ expected in vivo and the *k_x_* filtering effect of T_2_^*^ reduces the expected image quality when T_2_^*^ < *T_acq_*.

The in vivo *T_1_* 44 and T_2_^*^ mapping results add to the data in the literature for C_3_F_8_ in lungs. The *T_1_* of fluorinated gases has previously been attributed to have a direct correlation with ventilation‐perfusion.^34^, ^45^ Consequently, the differences in mean values for *T_1_* measured at 1.5T and 3T (Figure 5B) may be due to the level of saturation with the C_3_F_8_ + O_2_ mixture. T_2_^*^ correlates with lung inflation/filling level and may be related to alveolar size,^46^ which can change in diseases such as emphysema. Therefore, the parameter mapping techniques followed here may have direct relevance for future study.

Figure 2B demonstrates that at 1.5T using the optimal imaging parameters, the conservative SAR limits^38^ are exceeded because the optimal SSFP FA is high due to the near equivalence of *T_1_* and *T_2_* for the gases used in these experiments. Nevertheless, the sequence when run with a suboptimal FA of 72º still provides significant SNR gains over SPGR. However, at the higher field strength of 3T, SAR constraints are expected to further limit the potential advantage of SSFP for human ^19^F ventilation imaging. A prescribed *FA* during in vivo imaging at 3T of 30° was shown in phantom experiments (Figure 2A) to result in nearly the same SNR with SPGR and SSFP imaging.

There is a likelihood of some off‐resonance banding artifacts occurring in routine imaging, as may be observed near the diaphragm in some of the 1.5T SSFP images in Figure 7B and Figure 8A. Even in the geometrically uniform and relatively small cylindrical glass phantoms, banding can be observed at the susceptibility interfaces and as resonant frequency is offset (Figure 2A). Increased B_0_ and RF inhomogeneity, especially with FOVs as large as the human torso (38‐52 cm), increases this likelihood at the higher field strength of 3T. Previously, B_0_ mapping within the lungs with inhaled ^3^He gas at 1.5T and 3T demonstrated a variation in Larmor frequency at 3T of >120 Hz across the lungs.^47^ Therefore, the B_0_ inhomogeneity in the lungs makes the application less robust at 3T. Future investigations to test SSFP versus SPGR imaging at higher field strengths may show the expected improvement if the same imaging methods are reproduced in animal MRI where SAR limits are not exceeded and FA and B_0_ inhomogeneity can be reduced.

The expected SNR gains of using SSFP over SPGR imaging at 1.5T are comparable to the improvements seen with SPGR when going from 1.5T to the higher field strength of 3T. Therefore, equivalent quality human ventilation images may be obtained with the lower field strength without the same constraints of SAR. Especially, if at 1.5T a multi‐channel receive array is used as in this work and others.^5^ The use of a receive array for imaging of the thorax/torso may result in further SNR increases in the range of 50‐100%,^48^, ^49^ with the majority of the increase obtained at the periphery. Therefore, a mean SNR of 15‐20 may have been expected at 1.5T by combining the following factors: a measured SNR increase of 70% by use of SSFP, the approximate linear dependence of SNR with field strength,^50^ and the use of a receive array. We note that, at 3T k‐space was sampled with the use of an elliptical shutter where the corners of k‐space were not sampled (22% undersampling). Hence, despite the same nominal resolution of 1.5T and 3T for images in Figure 8 the SNR was slightly enhanced for the 3T images.

Here, the in vivo imaging at 1.5T was performed with a flexible vest coil,^42^ which typically would have a worse transmit homogeneity than rigid volume coils as demonstrated with direct comparisons with ^3^He hyperpolarized gas imaging at 1.5T in De Zanche et al^51^ (the variation was 7.3% within lungs with an asymmetric birdcage coil). Despite the lower frequency of 1.5T the flexible transceive array showed lower in vivo transmit homogeneity during in vivo imaging, while the transmit homogeneity with the birdcage coil at 3T was also not ideal (∼20% variation). The *FA* variation should not affect the in vivo T_2_^*^ parameter mapping, or the *T_1_* mapping because the colocalized FA maps were used in the fitting. The in vivo comparison of SPGR and SSFP imaging is confounded by the coil inhomogeneity (*FA* variation of ±22.4% in Figure 5A) and natural variation of T_1_ (24% in Figure 5B) and T_2_^*^ (28% in Figure 6B) throughout the lungs. These three factors lead to the range of variations in improvement with SSFP versus SPGR shown in Figure 7C and Figure 7F, and in the future may be investigated further.

Comparison of the in vivo ventilation image quality obtained here to previous studies is difficult due to differences in the imaging resolutions used and in the method of reporting and measuring SNR in images. Often, SNR is reported within a ROI with the highest signal. Further complicating the comparison, the longer $T_{\mathrm{aq}}$ used in previous studies results in broadened PSF as simulated in Figure 1D,E, which imparts a higher image SNR whilst degrading image quality due to blurring,^25^ and may be additionally modified by filtering during postprocessing.^14^ Additionally, different studies have used different RF transmit/receive coils that may contribute to more than a factor of 3 in SNR variation.

Nonetheless, in our study the measured SNR of 13.1 ± 5.7 throughout the lungs at 1.5T (8‐element transceive array with image resolution of 10 × 10 × 10 mm^3^ and $T_{\mathrm{aq}}=3.3ms$) is equivalent to the SNR of ∼30 reported by Gutberlet et al.^15^ (transmit birdcage and 16‐element receive array with image resolution of 7.8 × 7.8 × 20 mm^3^ and $T_{\mathrm{aq}}=7.1ms$). At 3T, the SNR achieved in our study of 11.6 ± 3.2 throughout the lungs (elliptical birdcage coil with image resolution of 10 × 10 × 10 mm^3^ and $T_{\mathrm{aq}}=2.1ms$) is also comparable to 32 ± 6 in a chosen central region reported by Couch et al.^14^ with a transceive vest coil and image resolution of 7.1 × 7.1 × 22 mm^3^ and $T_{\mathrm{aq}}=7.1ms$ with half‐fourier echo. Although the in‐plane resolution reported here at 3T is lower, visual comparison of the images in Figure 8 with those in Couch et al.^14^ show more clearly defined edges and features, similar to those obtained by Halaweish et al.^12^ at 3T, which did not report SNR values (with transceiver vest coil and image resolution of 6.25 × 6.25 × 15 mm^3^ and $T_{\mathrm{aq}}=7.7ms$).

The benefits shown here for SSFP of C_3_F_8_ are less applicable to the other common fluorinated gases of SF_6_ or C_2_F_6_ because of their shorter *T_1_* and *T_2_* values. Therefore, the use of C_3_F_8_ over other fluorinated gases has an increased benefit in terms of SNR achieved with SSFP and longer T_2_^*^. Consequently, the use of ultrashort *TE* (UTE) sequences for SF_6_ or C_2_F_6_ is logical,^14^ while not providing as dramatic an improvement for ^19^F lung imaging with C_3_F_8_ because T_2_^*^ is greater than gradient encoding and RF pulse times that may be used. Additionally, T_2_^*^ filtering in UTE SPGR imaging with fluorinated gases in 3D radial or 1D Cartesian UTE^11^ is another concern somewhat circumvented by the use of C_3_F_8_ with short TR SPGR or SSFP.

## 6 CONCLUSION

With optimized SSFP images we have demonstrated improved lung ventilation images with ^19^F C_3_F_8_ gas at 1.5T. We believe the image quality shown here to be equivalent or superior to images published previously at 1.5T or 3T and this work bodes well for the emergence of ^19^F gas MRI as a complementary modality to ^129^Xe or ^3^He MRI for directly imaging lung ventilation. However, benefits of SSFP for ^19^F C_3_F_8_ lung MRI at 3T are less clear.
